# Double polytetrafluoroethylene patch repair for diaphragmatic defect caused by diaphragmatic rupture following diaphragmatic resection with endostapler

**DOI:** 10.1093/jscr/rjae005

**Published:** 2024-01-24

**Authors:** Hiroaki Komatsu, Nao Furukawa, Hirotaka Kinoshita, Kazunori Okabe

**Affiliations:** Department of Thoracic Surgery, Bell-Land General Hospital, 500-3, Higashiyama, Naka-ku, Sakai-shi, Osaka 599-8247, Japan; Department of Thoracic Surgery, Bell-Land General Hospital, 500-3, Higashiyama, Naka-ku, Sakai-shi, Osaka 599-8247, Japan; Department of Thoracic Surgery, Bell-Land General Hospital, 500-3, Higashiyama, Naka-ku, Sakai-shi, Osaka 599-8247, Japan; Department of Thoracic Surgery, Bell-Land General Hospital, 500-3, Higashiyama, Naka-ku, Sakai-shi, Osaka 599-8247, Japan

**Keywords:** PTFE patch repairment, diaphragmatic defect, diaphragmatic rupture, diaphragmatic resection, diaphragm paralysis

## Abstract

A 41-year-old man developed phrenic nerve palsy after the resection of anterior mediastinal tumor, who underwent diaphragmatic resection with an endostapler. After the surgery, the surgical stump ruptured, resulting in a large diaphragmatic defect with the liver prolapsing into the thoracic cavity. Then, the diaphragmatic defect was closed with a polytetrafluoroethylene (PTFE) patch. The diaphragm was reconstructed using a second PTFE patch overlaying the diaphragmatic defect that had been closed by the first PTFE patch, because solely patching the diaphragmatic defect had a risk of recurrence of diaphragmatic elevation due to remaining original diaphragm and the presence of phrenic nerve palsy. The second PTFE patch was fixed to the lower ribs by non-absorbable suture. The postoperative course was favorable. After 3 months, his symptoms and pulmonary function improved. We underwent double PTFE patch repair in a patient with both huge diaphragmatic defect and phrenic nerve palsy.

## Introduction

Diaphragmatic plication is effective for unilateral diaphragm paralysis [1, 2]. However, diaphragmatic resection with an endostapler carries a risk of diaphragmatic rupture [3–5]. Few reports have focused on repair of a ruptured diaphragm staple line, and the most appropriate method of repair for a torn diaphragm is unclear. We herein report a case involving a patient who underwent double polytetrafluoroethylene (PTFE) patch repair for diaphragmatic defect caused by diaphragmatic rupture following diaphragmatic resection with an endostapler.

## Case report

**Figure 1:**
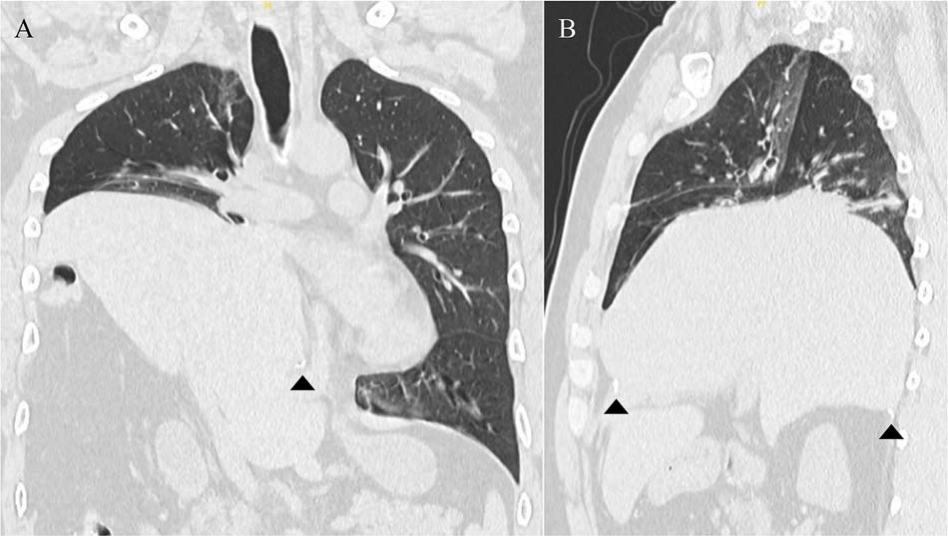
Chest computed tomography showing that the liver had prolapsed into the thoracic cavity. (A) Coronal view. (B) Sagittal view. The arrowhead indicates the edge of the torn diaphragm, including the torn staple line.

**Figure 2:**
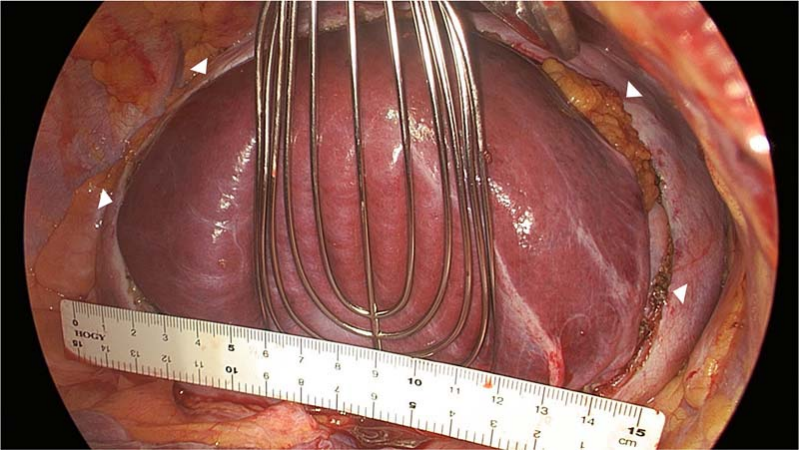
Surgical view showing the large diaphragmatic defect of 15 cm (arrowhead).

**Figure 3:**
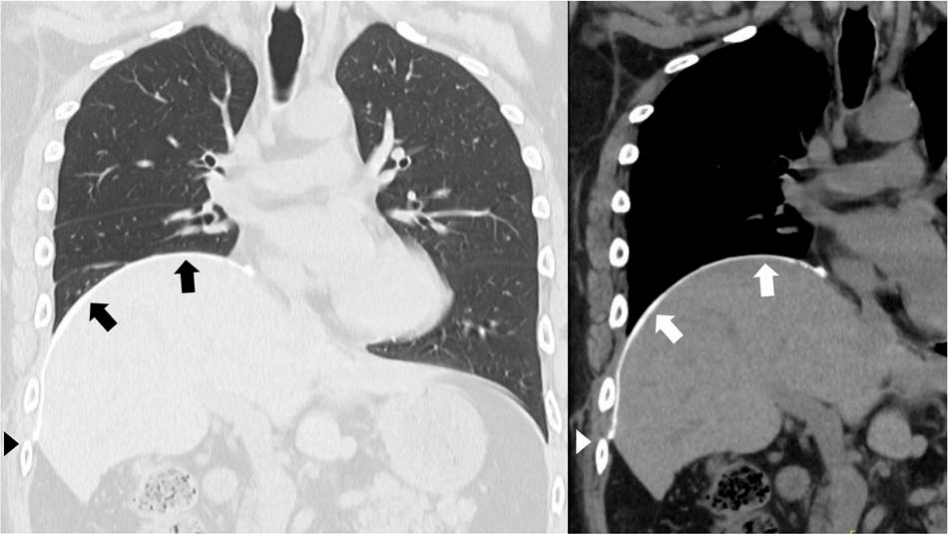
Postoperative chest computed tomography showing the reconstructed diaphragm using a second polytetrafluoroethylene (PTFE) patch above the original placement, overlaying the diaphragmatic defect that had been closed by the first PTFE patch (arrow). The second PTFE patch was fixed to the lower ribs (arrowhead).

A 41-year-old man was referred to our hospital because of chest pain. Chest computed tomography (CT) revealed an anterior mediastinal tumor, and it was resected by median sternotomy. Pathological diagnosis was thymic cyst. Postoperatively, the patient developed right phrenic nerve palsy due to intraoperative stretch of the nerve and dyspnea on effort. After a year, the phrenic nerve palsy didn’t improve. He then underwent diaphragmatic plication by a right mini-thoracotomy. The elevated diaphragm was resected using an endostapler (Powered Echelon Flex^®^, black and green cartridges) without any reinforcement. After the second surgery, the surgical stump left by the endostapler ruptured, resulting in diaphragmatic rupture and defect. Chest CT revealed a large diaphragmatic defect (arrowhead) and prolapse of the liver into the thoracic cavity (Fig. 1). However, this condition had been misdiagnosed as the recurrence of diaphragmatic elevation for a year. His dyspnea increased in the supine position, and pulmonary function testing revealed restrictive ventilatory impairment, which led the correct diagnosis of diaphragmatic rupture. The vital capacity (VC) and %VC were 1.86 L and 39.8%, respectively. A year after the second surgery, we then repaired the diaphragmatic defect by right thoracotomy. The surgical view of the thoracic cavity showed that the liver had prolapsed from the large diaphragmatic defect, which measured 15 cm (Fig. 2). Severe adhesion was present between the liver and the edge of the torn diaphragm including the torn staple line and was carefully dissected. The diaphragmatic defect was closed with a 1 mm thick PTFE patch. Moreover, the diaphragm was reconstructed using a second 1 mm thick PTFE patch above the original placement, overlaying the diaphragmatic defect that had been closed by the first PTFE patch (Fig. 3, arrow). The second PTFE patch was fixed to the lower ribs by non-absorbable suture (Fig. 3, arrowhead). The operating time was 325 min, and the blood loss volume was 50 mL. The postoperative course was favorable, and the patient was discharged 7 days after surgery. Three months after the third surgery, his symptoms improved, and pulmonary function testing revealed that the VC and %VC were 2.24 L and 48.1%, respectively. Postoperative chest computed tomography revealed PTFE patch maintained a good position (Fig. 3).

## Discussion

Several methods of surgical repair for diaphragm paralysis have been described, and diaphragmatic rupture after diaphragmatic resection with use of an endostapler has been reported [3–5]. The surgical stump left after diaphragmatic resection using an endostapler without suture reinforcement can easily rupture, and Kara *et al*. [6] recommended reinforcing the staple line by oversewing with nonabsorbable suture. In previous reports, the torn diaphragm was repaired by direct suturing [3, 4] or placement of a PTFE patch [5]. One patient who underwent direct suturing developed recurrence of diaphragmatic elevation [3], which was reconstructed with a PTFE patch. We believe that rupture of the staple line suggests that the thin diaphragm cannot continue to sustain the required tension. Therefore, repair by direct suturing is associated with a risk of re-rupture or the recurrence of diaphragmatic elevation. However, few reports have focused on repair of a ruptured diaphragmatic staple line. The most appropriate method of repairing a torn diaphragm after diaphragmatic resection using an endostapler remains unclear. In patients with a large hiatal hernia, PTFE patch repair results in less recurrence than does primary suturing [7]. In addition, pulmonary function gradually returns to preoperative levels in the long term after diaphragmatic plication [2]. Our patient was young and had a large diaphragmatic defect, and we performed PTFE patch repair with the expectation of a good long-term result. Moreover, simple patch closure of the diaphragmatic defect in this patient would have been associated with a risk of recurrence of diaphragmatic elevation because of remaining original diaphragm and the presence of phrenic nerve palsy. We thus reconstructed the diaphragm using a second PTFE patch above the original placement, overlaying the diaphragmatic defect that had been closed by the first PTFE patch. We have experienced good results of PTFE patch repair for diaphragmatic defects in patients who have undergone extrapleural pneumonectomy [8, 9]. In the same way, the second PTFE patch was fixed to the lower ribs, helping it to maintain a good position. After surgery, our patient’s symptoms and pulmonary function improved as expected.

In conclusion, we underwent double PTFE patch repair, consisting of first patch for the closure of a large diaphragmatic defect and second patch for the diaphragm reconstruction in a patient with both a huge diaphragmatic defect and phrenic nerve palsy. When the diaphragm is resected with an endostapler for the diaphragm paralysis, the staple line should be reinforced by nonabsorbable suture or other materials.
